# Nodulo-Infiltrative Subtype of Basal Cell Carcinoma With Follicular and Sebaceous Differentiation

**DOI:** 10.7759/cureus.107047

**Published:** 2026-04-14

**Authors:** Alyssa M Iurillo, Brian Chu, David Pomerantz, Jaclyn Anderson, Leslie Robinson-Bostom

**Affiliations:** 1 Dermatology, Indiana University School of Medicine, Indianapolis, USA; 2 Dermatology, The Warren Alpert Medical School of Brown University, Providence, USA; 3 Dermatology, School Street Dermatology, Pawtucket, USA; 4 Dermatopathology, The Warren Alpert Medical School of Brown University, Providence, USA

**Keywords:** basal cell carcinoma, dermatopathology, follicular differentiation, immunohistochemistry staining, nodulo-infiltrative subtype, rare variants, sebaceous differentiation, skin adnexal tumors

## Abstract

Basal cell carcinoma (BCC) is the most common cancer and typically presents as a slow-growing pearly papule with telangiectasias on sun-damaged skin, particularly the head and neck.Although BCC metastasizes rarely, delayed treatment can lead to significant local tissue destruction. Common subtypes of BCC include nodular, micronodular, superficial, and infiltrative. Rarer variants with sebaceous or follicular differentiation have also been reported.

We describe a case of a 69-year-old woman with a 7 mm red papulonodule on the upper back. Shave biopsy revealed nodulo-infiltrative BCC with sebaceous and follicular differentiation. Histopathology showed sebaceous cells with vacuolated, foamy cytoplasm expressing epithelial membrane antigen (EMA) and carcinoembryonic antigen (CEA). Follicular differentiation was characterized by infundibular cyst-like formations and cellular arrangements mimicking telogen follicles.

These rare subtypes, particularly in combination, pose diagnostic challenges. Increased awareness of their unique characteristics is essential to avoid misdiagnosis and ensure appropriate management. Further studies are needed to clarify their clinical behavior and outcomes.

## Introduction

Basal cell carcinoma (BCC) is the most common skin cancer, affecting one in five Americans [1]. BCC typically develops on sun-damaged skin, clinically presenting as pink pearly papules with telangiectasias. BCCs grow slowly, resulting in rare metastases, although they can be destructive and disfiguring if treatment is delayed [2-4].

Over two dozen subtypes of BCC have been reported in the literature. The most common clinicopathologic subtypes include nodular, micronodular, superficial, morpheaform, infiltrative, and fibroepithelial (also known as fibroepithelioma of Pinkus) [1]. These types can occur individually or in combination. The histologic subtype is an important distinction because certain growth patterns, such as infiltrative, morpheaform, and micronodular subtypes, are associated with increased risk of local recurrence and deeper invasion compared to nodular or superficial subtypes [1,3].

Follicular and sebaceous differentiation of BCC is rare. Follicular differentiation is characterized histologically by basaloid tumor nests with infundibular structures, keratin-filled cysts, and follicular germ-like arrangements resembling normal or telogen-phase hair follicles [5,6]. Sebaceous differentiation is defined by the presence of mature sebocytes or sebaceous duct structures within the basaloid tumor aggregates, which overlap with other sebaceous neoplasms [7-9]. When treated with routine excision, both these variants usually have favorable outcomes [6,7]. We present an unusual case of a nodular and infiltrative BCC with follicular and sebaceous differentiation.

## Case presentation

**Figure 1:**
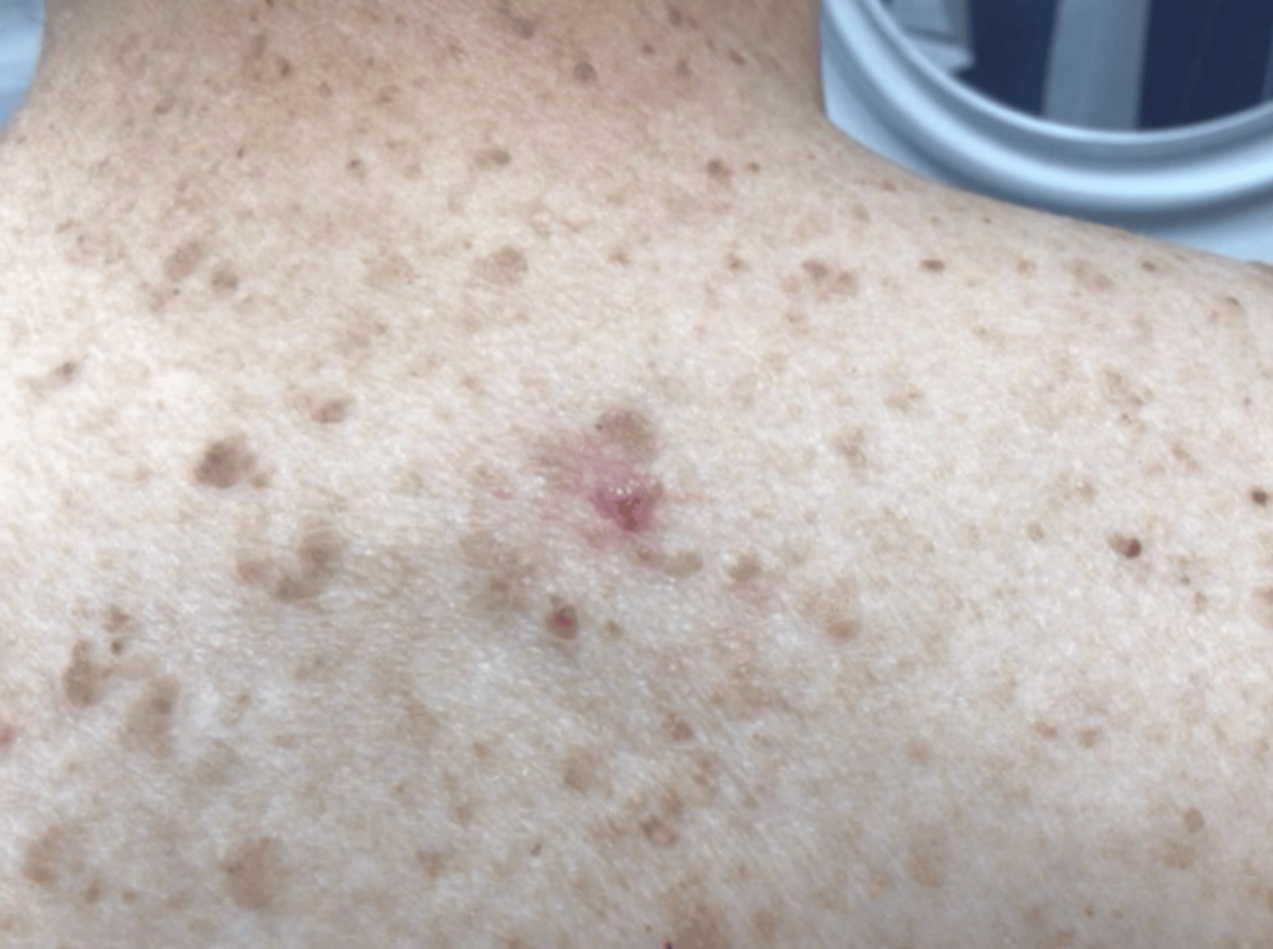
A 7 mm red papulonodule with overlying telangiectasias on the upper back.

**Figure 2:**
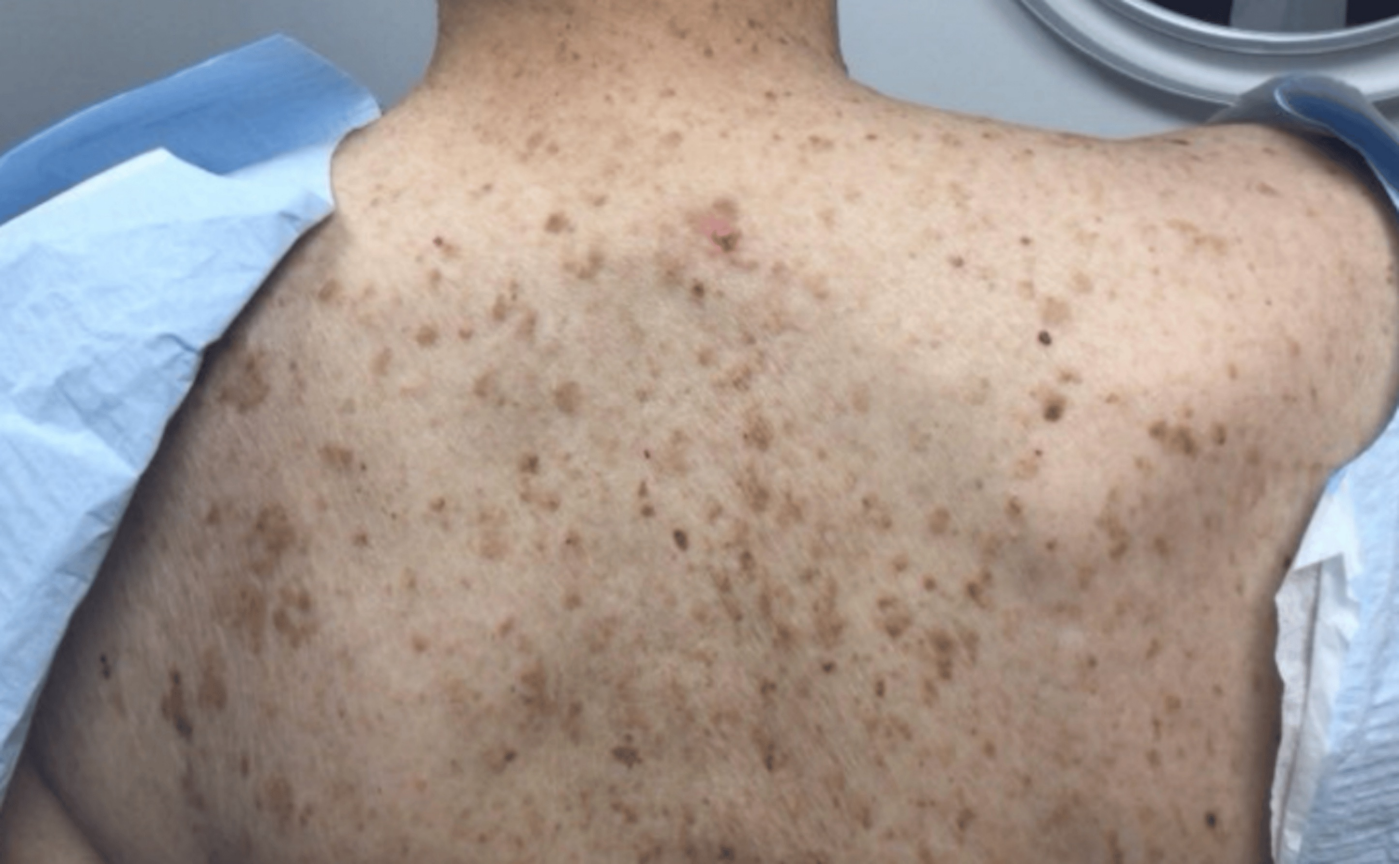
Wider field of view of the upper back with a red papulonodule.

**Figure 3:**
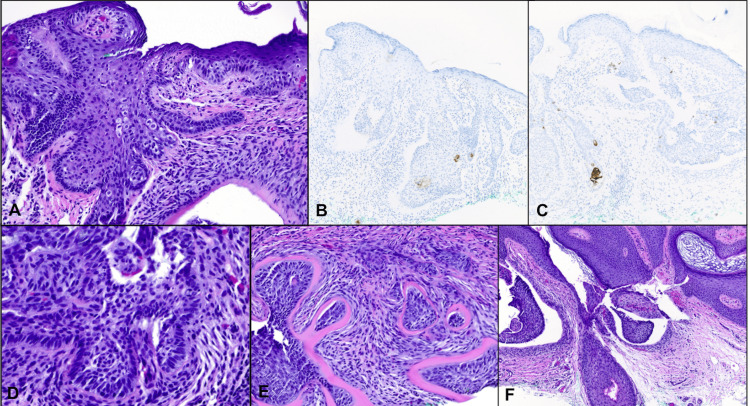
A. H&E sections showing sebocytes within the nodular basaloid islands extending from the epidermis, 200x. B. Immunohistochemistry for CEA highlights the sebocytes, 100x. C. Immunohistochemistry for EMA shows expression within the sebocytes, 100x. D. H&E sections with papillary mesenchymal-like bodies with peripheral palisading, 400x. E. H&E sections showing thickened basement membrane deposition around the basaloid islands, resembling a follicular cuticle, 200x. F. H&E sections showing the basaloid islands with a follicular structure with catagen-like appearance, 200x. H&E: hematoxylin and eosin, CEA: carcinoembryonic antigen, EMA: epithelial membrane antigen

A 69-year-old woman with previous melanoma in situ presented with a 7 mm red papulonodule with overlying telangiectasias on the upper back (Figures 1, 2). A shave biopsy revealed a nodulo-infiltrative subtype of BCC, with thickened basement membrane and exhibiting both follicular and sebaceous differentiation (Figure 3). Epithelial membrane antigen (EMA) and carcinoembryonic antigen (CEA) immunostaining confirmed the presence of sebaceous cells within the basaloid islands. The BCC was subsequently treated with routine excision without complication.

## Discussion

BCC is a keratinocyte carcinoma that can present with various patterns of adnexal differentiation, demonstrating its origin from basal cells of the epidermis and follicular unit. Although most BCCs are classified into common subtypes, such as nodular or superficial, rare variants with sebaceous or follicular differentiation present diagnostic challenges.

Unusually, our case demonstrated combined features of two rare histopathological BCC variants. Cases demonstrating both sebaceous and follicular differentiation are rare, and such overlap underscores the importance of careful histopathologic evaluation. BCC with sebaceous differentiation has been reported fewer than 10 times on PubMed and must be distinguished from other sebaceous tumors, such as sebaceoma, sebaceous adenoma, and sebaceous carcinoma [8,9]. Table 1 summarizes previously published case reports and case series of BCC with sebaceous differentiation. In addition to the typical findings of BCC, these tumors have areas of sebaceous differentiation with vacuolated cells containing foamy cytoplasm that express EMA, unlike typical BCCs [10]. The diagnostic criteria of this tumor vary between authors. BCC with sebaceous differentiation must be distinguished from sebaceous adenoma. BCC with sebaceous differentiation has a basaloid germinative cell component greater than 50% of the tumor lobules, with a round and slit-like reaction with mitoses and apoptotic debris. In contrast, sebaceous adenoma has a germinative cell lining with less than 50% of the diameter of tumor lobules [10]. Other sebaceous tumors include sebaceomas, which present as a haphazard array of germinative epithelium with sebocytes and sebaceous duct-like structures [10], and sebaceous carcinoma, which presents with pagetoid spread in the epidermis, desmoplastic stroma reaction, haphazard infiltrative growth, and invasion of nearby structures [4,8,11-13]. The rarity of BCC with sebaceous differentiation may be due to unclear nomenclature surrounding sebaceous neoplasms and limited awareness of this variant. BCC with sebaceous differentiation usually has a favorable outcome when properly treated with a low occurrence of adverse prognosis [7].

**Table 1:** Previously published case reports/series: BCC with sebaceous differentiation. BCC: basal cell carcinoma, EMA: epithelial membrane antigen, HTN: hypertension

Case (author/year)	Age/sex/demographics	Location (site)	Lesion size	Histopathology key findings	Treatment and outcome
Save et al. 2018	42-year-old man, hypertensive, otherwise healthy	Left nasolabial fold	1.0 × 1.5 cm	Poorly circumscribed dermal tumor, basaloid cells with peripheral palisading, retraction artifacts, sebaceous differentiation with sebocytes	Surgical excision with 5 mm margin, clear margins, no recurrence at two years
Misago et al. 2004	72-year-old woman	Right ala nasi	1.4 x 1.1 cm	Typical BCC features with duct-like structures and vacuolated cells with foamy cytoplasm, indicative of sebaceous differentiation, EMA positive	Completely excised, and a nasolabial pedicle graft was applied. No evidence of recurrence was observed in the subsequent one-year period
Shash et al. 2020	81-year-old woman with HTN, osteoporosis, and rheumatoid disease	Left nasal ala	0.6 × 0.5 × 0.5 cm^3^	Dermal lesion with basaloid cells with peripheral palisading, focal stromal clefts, and scattered sebocytes	Mohs surgery and full-thickness skin graft, recurrence at four months managed by wide excision with 4-mm margins, ongoing follow-up with no recurrence at ~18 months
Akhtar et al. 2016	42-year-old man	Right cheek	1.5 × 1.5 cm (ulcerated nodular)	Basaloid nests with peripheral palisading and sebaceous ductoid structures with vacuolated sebocytes	Complete excision, asymptomatic at one-year follow-up

BCC with follicular differentiation (or infundibulocystic BCC) mimics other follicle-derived neoplasms [6]. Table 2 summarizes the previously published case reports and case series of BCC with follicular differentiation. This variant is characterized by follicular structures within the tumor, such as infundibular cyst-like formations and cellular arrangements mimicking telogen hair follicles [5]. Small cyst-like formations contain cornified cells and clefts within the stroma itself, compared to typical BCC that has clefting artifacts between aggregations of neoplastic cells and stroma [5]. When compared to benign adnexal neoplasms, the epithelial aspect of BCCs is much more striking than the connective tissue aspect [5]. Follicular differentiation can present with embryonic follicular germs, authentic follicular germs, small infundibular cysts, matrical cells, areas stimulating hair follicles in anagen, the inner root sheath of the hair follicle, or trichilemmal differentiation [6]. Infundibulocystic BCC tends to be small, with a low tendency to infiltrate or ulcerate [6].

**Table 2:** Previously published case reports/series: BCC with follicular differentiation. BCC: basal cell carcinoma

Reference (first author/year)	Age/sex/demographics	Site of lesion	Size	Histopathology/follicular features	Treatment and outcome
Tozawa and Ackerman, 1987 (15 specimens, series)	Mixed (53-84 years (median age in the mid-60s), with a slight female predominance (eight females, seven males)	Face (various facial sites) Lesions involved sun-exposed facial sites, including the cheek, chin, face, eyelid, eyebrow, ear, nose, lip, and periauricular region.	Small (less than 5 mm) circumscribed well (specifics not reported)	Numerous tiny infundibular cyst-like structures and neoplastic basaloid cells resembling follicular elements, classic description of follicular differentiation variant	Surgical excision or Mohs; small, indolent BCCs with low aggression reported; details by case variable. See the article for details.
Sánchez Yus et al., 1989, Case 1	62-year-old woman	Nasolabial fold	7-mm round lesion with pearly borders, present for ~eight months	BCC with advancing borders and central regression, tumor nodule in continuity with a follicular infundibulum, and an infundibulum with comedo-like dilation. The deep portion showed a follicular bulb with a wide papilla formed by tumor cells, a prominent basement membrane, and central necrosis	Surgical excision
Sánchez Yus et al., 1989, Case 2	74-year-old man	Retroauricular sulcus	Small lesion on the retroauricular sulcus (size not specified)	BCC partly undifferentiated and partly showing follicular differentiation with formation of a follicular bulb and small papilla; bulb with outer root sheath, central keratinized inner root sheath, and hair shaft; follicle dilated into BCC mass	Surgical excision
Kato and Ueno, 1993	56-year-old woman	Left nasal bridge	2 × 4 mm	Superficial, well-circumscribed basaloid tumor with multiple infundibulocystic structures, cysts lined by infundibular epithelium containing cornified cells, basaloid-to-squamous infundibular differentiation with follicular germ-like buds, alcian blue-positive mucinous cyst contents, no follicular bulbs or papillae	Surgical excision under local anesthesia, no recurrence at 10-month follow-up
Herman et al., 2003 (multiple unilateral lesions)	83-year-old woman	Right perioral region (multiple papules)	3- to 5-mm papules were located along the right nasolabial fold, right upper and lower cutaneous lip, and right chin	Infundibulocystic BCC on biopsy: small, basaloid neoplasms in superficial dermis, composed of anastomosing cords and strands of basaloid keratinocytes with multiple infundibulocystic structures. Focal peripheral palisading and rare apoptotic bodies were present	Given the multiplicity of lesions and their cosmetically sensitive perioral distribution, a conservative management approach was selected. Lesions remained clinically stable for over a decade, and ongoing photographic surveillance was pursued with surgical intervention reserved for lesions demonstrating change or progression.
Toyoda et al., 1998	72-year-old woman	Right preauricular region	1.2 × 0.7 cm	Numerous infundibular cysts lined by follicular infundibular epithelium, containing cornified cells with scant keratohyalin. Bud-like projections from epithelial cords simulated follicular germ differentiation, with peripheral palisading of tumor nests.	Surgical excision under local anesthesia, no recurrence reported; the article does not specify the long-term follow-up duration
Kawasaki et al., 2018 (infundibulocystic BCC mimicking nevus)	52-year-old woman	Left temporal scalp	1.5 cm	Solid and funicular fascicles of squamoid cells with multiple keratinous (epidermal) cysts. Focal peripheral palisading, mucinous stromal deposition, and absence of lower follicular structures (follicular bulbs or papillae) supported the diagnosis of infundibulocystic basal cell carcinoma.	Surgical excision with local anesthesia, with a focally positive deep margin, a second excision was performed, showing a more aggressive growth pattern than typically described for infundibulocystic BCC.

Previously published case reports of BCC with sebaceous differentiation and BCC with follicular differentiation can be found in Tables 1-2, respectively [14-20].

This coexistence of sebaceous and follicular differentiation in our case uniquely highlights the range of adnexal differentiation that BCC can present with. Acknowledgment of this overlap is important because of the possibility of misclassification as a primary sebaceous neoplasm or benign adnexal tumor, which can lead to inappropriate workup for syndromes or overly aggressive treatment. Although these variants have unusual histology, BCC with sebaceous and follicular differentiation generally show indolent behavior and good outcomes when conservatively treated.

## Conclusions

Comprehensive studies or case reports addressing BCCs with combined follicular and sebaceous differentiation are limited. This underscores the need for further research to understand the clinical behavior, prognosis, and best management of such rare presentations. Given the infrequency of this combined differentiation, pathologists and clinicians should be aware of the potential for such occurrences to ensure accurate diagnosis and appropriate treatment planning. BCC with sebaceous or follicular differentiation follows a similarly favorable clinical course as other subtypes when properly treated.
